# Litigation-Generated Science: Why Should We Care?

**DOI:** 10.1289/ehp.9987

**Published:** 2007-11-07

**Authors:** Leslie I. Boden, David Ozonoff

**Affiliations:** Department of Environmental Health, Boston University School of Public Health, Boston, Massachusetts, USA

**Keywords:** biomedical research, conflict of interest, *Daubert*, litigation, peer review, regulatory science, science and litigation, scientific evidence

## Abstract

**Background:**

In a 1994 Ninth Circuit decision on the remand of *Daubert v. Merrell Dow Pharmaceuticals, Inc.,* Judge Alex Kosinski wrote that science done for the purpose of litigation should be subject to more stringent standards of admissibility than other science.

**Objectives:**

We analyze this proposition by considering litigation-generated science as a subset of science involving conflict of interest.

**Discussion:**

Judge Kosinski's formulation suggests there may be reasons to treat science involving conflict of interest differently but raises questions about whether litigation-generated science should be singled out. In particular we discuss the similar problems raised by strategically motivated science done in anticipation of possible future litigation or otherwise designed to benefit the sponsor and ask what special treatment, if any, should be given to science undertaken to support existing or potential future litigation.

**Conclusion:**

The problems with litigation-generated science are not special. On the contrary, they are very general and apply to much or most science that is relevant and reliable in the courtroom setting.

“Litigation-generated science” came to center stage in Judge Alex Kosinski’s 1995 decision in the Ninth Circuit remand of *Daubert v. Merrell Dow Pharmaceuticals, Inc*. There, Kosinski asserts

that the testimony proffered by an expert is based directly on legitimate, preexisting research unrelated to the litigation provides the most persuasive basis for concluding that the opinions he expresses were ‘derived by the scientific method.’ (*Daubert v. Merrell Dow Pharmaceuticals, Inc.* 1995)

Conversely, he indicates that research undertaken to support litigation should bear a special burden to demonstrate its admissibility. Here, Kosinski uses a narrow definition of litigation-generated science to refer to studies that begin after litigation is initiated (and probably after the expert is hired) and is funded by one of the parties or their attorneys. The contrast is with

experts whose findings flow from existing research [who thus] are less likely to have been biased toward a particular conclusion by the promise of remuneration; when an expert prepares reports and findings before being hired as a witness, that record will limit the degree to which he can tailor his testimony to serve a party’s interests. (*Daubert v. Merrell Dow Pharmaceuticals, Inc.* 1995)

Judge Kosinski implies that experts conducting research to support litigation do not follow the norms of science, and that they are at high risk of engaging in scientific misconduct or even fraud. Implicitly, his view is that expert witnesses are more “willing to say whatever is needed to advance the cause of the party that hires them” (Haack, in press).

In singling out scientific testimony for enhanced “gatekeeping” requirements by the trial court, the *Daubert* opinion noted the words of Judge Jack Weinstein about the power of scientific authority and its potential to mislead:

Expert evidence can be both powerful and quite misleading because of the difficulty in evaluating it. Because of this risk, the judge, in weighing possible prejudice against probative force under R.403 of the present rules, exercises more control over experts than over lay witnesses. (*Daubert v. Merrell Dow Pharmaceuticals, Inc.* 1993)

The argument is that research done for a specific case is especially subject to manipulation but that the evidence is both difficult for juries to evaluate and unusually persuasive. On the surface this seems reasonable, or at least plausible, but a hard look at the question of litigation-generated science makes some of the easy distinctions problematic.

The question comes to us in the context of the judicial landscape after the *Daubert* decision and its successors, *Kumho* and *Joiner* (Berger 2005). Since the 1993 *Daubert* decision, federal judges (and state judges where individual states have followed the *Daubert* pattern) are required to give more careful scrutiny to the relevance and reliability of adversarial expert evidence. Empirical evidence is scanty, but many observers believe that after *Daubert* the parties in civil cases have had more difficulty getting their scientific evidence heard by juries (Berger 2005; Dixon and Gill 2001; Krafka et al. 2002). If true, this places a special burden on plaintiffs, who have an affirmative requirement to demonstrate their case, which can be dismissed on summary judgment if their evidence is excluded.

How well *Daubert* serves the goal of justice has been hotly debated. This present article does not join that debate. Rather, we focus on the narrower question of whether, under *Daubert* or any other framework, litigation-generated science should be treated differently from other science offered as evidence in the courtroom. We also address a boundary question: What science is litigation generated, and what science is not, or, indeed, does the boundary itself make any sense?

Our analysis begins with a discussion of science done explicitly to support litigation. We contend that concern about this science is largely because of the financial incentives of scientific experts to obtain results that support the position of the parties that hired them. We then show that this concept of interest applies to a much larger set of studies, many of which are not explicitly litigation related and are done before any litigation commences. We also address the argument that litigation-generated research has not been peer reviewed and is therefore less trustworthy. We contend that peer review, although an important and useful tool for improving scientific publications, has significant limitations when used by judges to determine the reliability of scientific studies. And we suggest that cross-examination by attorneys aided by competent experts may serve the ends of justice at least as well as peer review. We conclude there is not a sufficient case for treating certain kinds of litigation-generated science differently.

## Litigation-Generated Science, Type 1

We begin with two questions about Judge Kosinski’s definition of litigation-generated science—research undertaken to support litigation—which we will call litigation-generated science, type 1 (LGS1). First, we ask if there is any evidence that LGS1 is less reliable than research done before litigation. We can quickly dispose of this question because we are aware of no empirical research that demonstrates this and certainly none that would survive a *Daubert* hearing on this issue. It is true that authors have offered anecdotal examples of good and bad litigation-generated science, but there are no rigorous analyses comparing the quality of studies done for litigation with other studies.

Second, are there any relevant distinctions between LGS1 science and other science? In our context we sharpen this question by asking if the incentives to do biased studies are qualitatively different for LGS1 science compared with those for non-LGS1 science. Stated differently, if an expert has done research to support a court case either as an employee or as a hired consultant, to what extent should a judge be concerned about the expert’s bias, and how it might affect the quality of the study beyond that of any other scientist?

On its face, doing science for the purpose of supporting one side or another in a legal proceeding would seem to have built-in incentives to “paint the target around the arrow,” and there is little doubt this happens with some LGS1, as it does for testimony about non-LGS1 science. Indeed, this potential exists for almost any testimony, even that of fact witnesses. But Judge Kosinski further seems to assume that science done before litigation is not subject to bias because it is science done without knowing what the “right answer” should be. We know this blank slate view of scientific practice is not strictly true because scientists’ attitudes and interests can affect their research, so Judge Kosinski implicitly suggests that any motives other than money have relatively small effects or work equally on both sides and thus “cancel out.”

### Follow the money?

What kind of incentives or disincentives might be present for a scientist doing LGS1 that would be absent in other settings? The obvious and primary one is money. Experts who do research and testify in court cases are typically well compensated. These economic incentives can be quantified, which allows the strength and nature of the incentive to be more easily understood than other kinds of motivators (e.g., academic promotion, professional reputation, personal attitudes). Often it is not just the lump sum involved with a professional fee or salary that is the economic incentive. Future business or continued employment may be even more powerful. The fact that these incentives (and their complementary disincentives) are widely shared and experienced gives psychological cogency to the economic dimension as a problematic aspect of litigation-driven science and expert testimony. Buttressing the economic dimension, the social relationship between funder and scientist can even affect the attitudes of scientists who seek to maintain a disinterested perspective. Moreover, this may occur without any consciousness of bias on the part of the expert (Freudenberg 2008). This problem also exists for any employee of a company who does science on its behalf.

Various departures from the norm, such as fraud, data falsification, selective use of data, and the systematic skewing of interpretations, all occur to some extent outside the courtroom, too. But in what way do economic incentives specifically make a scientist more likely to act in a manner that violates the usual norms of science? Someone who has a valuable product is also less likely to spoil the brand by providing unsatisfactory merchandise (as seen by the client). Scientists hired to support litigation often command high fees. Employee scientist witnesses have a different but equally obvious problem: It is not their fee but their whole livelihood that might be at stake.

### Conflicts of interest in science

All this suggests that LGS1 boils down to a special case of science funded by parties with an interest in the outcome—conflicts of interest in science (Krimsky 2003, 2005). We can reframe the LGS1 issue in this way: A researcher who conducts a study funded by a party in a court case has a financial conflict of interest, and the court’s concern is that this conflict will lead the researcher to conduct the study or interpret the results in a manner designed to suit that party. If this is the reasoning that underpins the extra *Daubert* hurdle for LGS1 studies, we need to ask if other (non-LGS1) studies are immune from the same concerns. We think not.

A growing body of scholarship has consistently raised concerns about bias generated by conflicts of interest outside of LGS1, for example, safety and efficacy studies funded by pharmaceutical companies. Overall, compared with studies by researchers without financial conflicts of interest, such studies have been found to have conclusions more favorable to the companies funding them (Als-Nielsen et al. 2003; Bekelman et al. 2003; Friedman and Richter 2004; Lexchin et al. 2003; Perlis et al. 2005; Stelfox et al. 1998). In addition, biomedical industry relationships are associated with delay in reporting research results (Blumenthal et al. 1997, 2006). When these delays are caused by proprietary or trade secret concerns, they also violate scientific norms.

The financial conflict of interest in these allegedly nonlitigation studies is clear. Approval of new drugs can literally add billions of dollars to annual profits. To obtain approval, companies must demonstrate safety and efficacy. Failure to show either can lead the U.S. Food and Drug Administration (FDA) to delay or ultimately deny approval. Thus, the companies funding drug trials obviously need and want results that support their applications. This research is begun well in advance of any possible liability litigation and not explicitly to support a position in a lawsuit. But such research purporting to demonstrate safety could be used later by companies defending themselves against suits by individuals who allege they were injured by the pharmaceutical in question and, although not LGS1 by Judge Kosinski’s definition, serve the same purpose and work in the same way. Scientists and companies are aware of this aspect of safety research conducted before a product hits the market (Haack, in press).

A similar example is found in studies to support U.S. Environmental Protection Agency (EPA) premarket registration or reregistration of pesticides. Here, as in the FDA case, manufacturers fund safety studies even though there is a clear conflict of interest. The U.S. EPA does not object and even requires these studies. As with pharmaceuticals, safety studies are also available to defend liability lawsuits, and manufacturers and consultant or employee scientists are aware of this. Similarly, chemical manufacturers conduct research in support of the safety of their products. Even when these products do not require licensing, research can satisfy the safety concerns of purchasers and, again, can prove valuable if liability litigation should ensue (or dangerous if they reveal evidence of a hazard).

Science can also be done with litigation in mind but not connected to a particular case, requirement, or licensing application. Large corporations often invest strategically in research agendas whose objective is to develop a body of scientific evidence favorable to a particular economic interest or useful for defending against particular claims of legal liability (see “LGS2: Strategic Science and Manufactured Doubt”). The use of “neutral” trade associations or for-hire industrial research laboratories has a long history that has been detailed in other published articles (Michaels and Monforton 2005; Ozonoff 1988; Proctor 1996).

Direct funding of a specific study by an interested party is not the only dimension of financial conflict of interest. Financial conflicts can be generated by funding of other studies, research-related gifts, board membership, and stock ownership. Indeed, studies of physician behavior show that payments for meals, conference travel, and continuing education affect physician prescribing practices (Wazana 2000). It is not clear why direct funding by interested parties of prelitigation studies or these other conflicts of interest differ qualitatively from LGS1.

In addition, there are some areas for which virtually all research presented in court is LGS1. When an employer is charged with a pattern of workplace discrimination, the most convincing evidence is a statistical study of hiring, termination, promotion, and wage patterns. Such a study would virtually never be undertaken before discrimination had been alleged. If it were difficult to use such studies in litigation, plaintiffs claiming discrimination would be greatly disadvantaged.

As a final observation about what is different about LGS1, we note there are certain types of expert testimony employing scientific methods that are done routinely only for court cases. Accident reconstruction is a prime example. And there are some allegedly scientific methods that have been researched and designed to be used almost exclusively by one side in a legal setting, for example, fingerprint analysis. Because of the specificity of these studies, many might not consider them “scientific,” although they use all the same techniques and modes of reasoning. The only difference is that they have restricted generalizability. But the same is true of studies using scientific methods done expressly for a single case. Why should LGS1 studies be any more objectionable than accident reconstruction or fingerprint identification? There is good reason to argue that, for example, forensic testimony, is no less litigation related and potentially at least as prone to conflicts as a study that meets the requirements of Judge Kosinski’s definition (Jasanoff 2006; Risinger and Saks 2003). Indeed, the *Kumho* decision, extending the reach of the *Daubert* ruling to all expert testimony based on “technical” or “other specialized knowledge” (*Kumho Tire Co. v. Carmichael* 1999), explicitly acknowledges this.

## LGS2: Strategic Science and Manufactured Doubt

We return now to other kinds of “litigation-driven science,” science done to establish the safety of a specific substance, product, or procedure; science done with the strategic aim of spoiling science that might at some future point become inconvenient; or science done to establish results that might be useful against a competitor (e.g., in patent infringement or market share). We refer to this as LGS2.

All the incentives and conflicts of interest we discussed with LGS1 pertain here, as well. A major difference is one of time scale. LGS1 usually has a time-constrained application, although given the drawn-out nature of legal battle today, even this distinction does not always apply. A second difference is that LGS2 may have more than one goal. Its explicit goal may be to support FDA approval of a drug or to support claims of safety for an industrial or consumer product. But, it is easy to see that evidence of safety in support of a marketing effort can be used later to defend against claims of harm. We can think of both LGS1 and LGS2 as subsets of strategic science.

One can even make the argument that LGS2 is even more likely to mislead than LGS1. Well-financed industries have the resources to seed the literature with strategic science. Take, for example, a line of argument designed to show that certain kinds of chemically induced animal tumors are not relevant to human risk assessment (Melnick et al. 1996, 2008). This can be used to make a case that such animal studies should be excluded. There is a covert litigation-driven relationship between LGS2 and the general literature that is currently less likely to be subjected to the same additional scrutiny routinely applied to science that is explicitly case specific.

This raises another important issue: potential defendants in toxic tort litigation (and law enforcement officials on the criminal side) have the resources to conduct or fund research valuable for litigation that has contingent value, but potential civil plaintiffs (and criminal defendants) do not. Although a few plaintiffs (or defendants in criminal trials) might avail themselves of LGS1, the option of prelitigation research (LGS2) is closed to them. The litigation setting may be the only venue in which civil plaintiffs have access to science done on their behalf. Even worse, in many cases, the vast majority of—or all—research on a product’s hazards may be conducted under the sponsorship of its manufacturers or by researchers with industry ties. If the legal system treats LGS1 as highly suspect but treats prelitigation research as without conflict, it places its thumb on the scales of justice.

Much of the research that characterizes hazards is funded by the interested parties, because they have the resources, access, obligation, and expertise to conduct this kind of research. Indeed, a recent study of biomedical research shows that 57% of biomedical research funding in 2003 came from industry, compared with 28% from the National Institutes of Health (Moses et al. 2005). Nearly $100 billion was spent on biomedical research in 2003 (Moses et al. 2005), and expenditures have certainly surpassed that amount by now. Although there are no data on this, it is plausible that funding for plaintiff-oriented research is a very small proportion of the total. Setting hurdles before such science—science that is indeed required in many regulatory proceedings—would seem both undesirable and counterintuitive (Henry and Conrad 2008). Exclusion or a rebuttable presumption against both LGS1 and LGS2 would have a disproportionately negative impact on plaintiffs by excluding much of the available evidence. With no evidence to present to the jury, the plaintiff cannot prevail. Moreover, the arguments presented against LGS1 apply with equal force not only to LGS2 but also to all expert testimony. The Kosinski remedy implies that experts hired by any party to a dispute often are not reliable and should be excluded.

Of course, LGS1 produces scientific results obtained specifically for a case. Perhaps the questions, observations, and methods would thereby be subject to bias or manipulation in a way that would allow the testifying expert to build a stronger case. On the other hand, when testifying about the state of preexisting scientific literature or standards of practice, this extra degree of freedom is removed. However, it could also be argued that there is more freedom to interpret science not specifically tied to the facts of a particular case. Cherry-picking the literature is one manifestation of this, but it is not uncommon for two scientists to interpret the same study very differently. And the litigation setting can both uncover and highlight such differences. Conflicting expert interpretations of scientific studies in the courts and in regulatory settings remain the rule not the exception. In most cases, the studies under question were not done expressly to support a party to litigation (Jasanoff 1990, 1996). We lack data about whether LGS1 or more general studies provide greater latitude for interpretation, so these speculations remain unverified.

The antidote to either LGS1 or LGS2 is not to use the litigation motive as a blunt instrument for exclusion but as a commonsense argument for expanded discovery and greater latitude for cross-examination by the parties. This is particularly true for LGS2, where company motives that appear unrelated to the case at hand may be highly pertinent. This clearly presents itself in *Blum v. Merrell Dow Pharmaceuticals* 1997, elegantly presented and discussed by Susan Haack (in press).

## The Role of Peer Review and Publication in Scientific Journals

One argument for excluding LGS1 studies is that the specific nature and shorter time frame make them less likely to undergo the usual conventions of scientific peer review. Peer review can provide a check on science that is poorly designed, does not conform to established conventions, adopts arbitrary methods, or is poorly written. Such science is less likely to be accepted in peer-reviewed journals. Peer review is also important in encouraging scientists to refine underlying models and arguments. Still, we know that peer reviewers frequently disagree about whether to accept papers (Rothwell and Martyn 2000). We also know that poorly designed and analyzed studies can easily receive favorable reviews (Curfman et al. 2006; Smith 2006). Indeed, many published articles undergo only the most cursory peer review (Jasanoff 2008). Either via poor judgment or conscious intention, scientists choose study topics, opt for study designs, do analyses, and interpret results in ways that bias conclusions in one direction or another (Melnick et al. 2008). Sometimes, peer review will reject such studies, but often it will not. In addition, unlike the idealized image of peer review, the decisions of peer reviewers often do not determine publication. Editors always have the final say, so well-reviewed articles may not be published, whereas at the same time, editors approve publication of poorly reviewed studies (Jasanoff 1990). Moreover, although peer review may expose weak study design or lapses in a scientist’s understanding or logic, it is unlikely to detect any but the most blatant fraud or scientific misconduct (Smith 2006). Here we differ with the idealized picture of peer review presented by Henry and Conrad (2008). Peer review is not a remedy for Judge Kosinski’s concerns [see also Jasanoff (1990)].

Legal cases sometimes engender research to fill a void in scientific knowledge or to answer questions specific to a given setting. In such cases, the peer review process may be too slow and cumbersome to provide timely information to the legal system. Alternatively, research appropriate to answer factual questions critical to a legal case may be too narrow to warrant peer-reviewed publication, even if its methods are impeccable. In other cases, innovative methods may be exactly the type needed to answer questions of fact raised in litigation, although they may fare poorly in peer review that rewards “inside the box” thinking and penalizes the new idea or method. Many classic articles in economics, including seminal articles that eventually led to the awarding of Nobel Prizes, had great difficulty being accepted for publication (Gans and Shepherd 1994). These points are recognized in the *Daubert* opinion (*Daubert v. Merrell Dow Pharmaceuticals, Inc.* 1993):

In some instances well-grounded but innovative theories will not have been published. . . . Some propositions, moreover, are too particular, too new or of too limited interest to be published.

Finally, peer-reviewed publication can be manipulated by the parties to litigation. It can “fall apart if lawyers and litigation experts invade the realm of scientific research and manipulate the medical and scientific publication system to achieve their litigation ends” (Anderson et al. 2001).

Few, if any, journal peer-review processes are as stringent or as probing as the usual cross-examination performed in an adversarial setting. It is simply not true that LGS1 studies do not undergo peer review. Lawyers routinely hire consultants to go over the minutiae of any study offered up by the other side. Alleged study flaws are then used in cross-examination to devalue or deconstruct the study in the eyes of the jury.

We do not claim that peer review and cross-examination are interchangeable. Each is based on different assumptions, and each operates in a different manner. Peer review typically assumes that the submitted article is an accurate representation of the underlying research and that the research itself is honest and not intentionally biased or misleading. Peer review is a filter, designed to let through research that is original and significant, as well as based on good data and a valid research design. It also serves the functions of improving analysis and clarifying exposition. Overall, peer review is designed to improve the quality of the scientific literature. Conversely, cross-examination assumes that research is designed to buttress the opponent’s position and is both biased and misleading and probably poorly designed as well. Cross-examination is designed to deconstruct and undermine the credibility of an adversary’s expert testimony or research, not to improve its quality (Jasanoff 1992). By taking an adversarial stance, cross-examination may reveal hidden assumptions and errors not uncovered by peer review (Jasanoff 1996).

Given these differences, cross-examination may be a better tool than peer review to expose purposefully misleading research. A competent attorney, aided by competent experts, should be in a better position to expose the flaws in such research than is the peer reviewer, who often takes less time than the expert in a legal case and has more limited resources to probe than does the cross-examining attorney (Jasanoff 1996).

Perhaps the strongest argument related to peer review derives from the fact that LGS1 is typically unpublished and therefore not exposed to the scrutiny of the scientific community. One aspect of such scrutiny is that publication may lead to new research that contradicts the original findings. Perhaps more important is that publication allows scientists’ work to be read by their scientific peers. Scientists do not want their peers to read their badly flawed or, even worse, dishonest research. So, it may be possible that they are willing to engage in such research within the confines of a trial but would be unwilling to submit it for publication. This is an argument against all unpublished research, not just LGS1, and it is also an argument against all adversarial experts, not just scientists testifying about LGS1.

This then comes back to the question of the effectiveness of cross-examination in revealing to the jury the nature of poorly done and deceptive research. Cross-examination by attorneys who have been briefed by their own experts can accomplish the task and is potentially more useful than conventional publication peer review.

## LGS1 and LGS2 and the Objective of the Research Endeavor

We have already noted that a possibly relevant difference between case-specific scientific evidence and more general evidence of science is the purpose for which it is done. Presumably, the goal of non-case-specific science is less applied and more general.

But a great deal of “normal” science is quite applied, especially in the area of toxicology and epidemiology. This is true in particular for all regulatory science. Experiments or observational studies of chromium exposure are not done for the purpose of disinterested knowledge but because knowledge of the health effects of chromium is important and relevant for practical purposes—to protect workers and the general public from harm. To the extent that the implications are evident to all concerned (the scientists, research funders, readers), there seems to be little difference regarding the objective. In the case-specific instance, there may be tighter constraints that answer narrower questions because generalizations to other populations are not as important, but this is a matter of degree, not of kind.

Whether it is LGS1, LGS2, or neither, the desire to have the expert “just be a scientist” is doomed to failure. Science (litigation generated or not) is presented at trial by partisan expert witnesses. These experts use scientific evidence to advocate for a position and, in the process, transform it into a tool of advocacy. Even when the science is the same, what Jasanoff (2008) calls the “argument and representation” are different. As it is presented at trial, even normal science is integrated into an advocacy narrative and becomes unmoored from the discourse of science as practiced outside the litigation context. The usual rhetoric of science is displaced by the rhetoric of courtroom testimony that separates the common “on the one hand, on the other hand” narrative by a single scientist to one where the “two hands” are represented by two different experts. Even before any evidence is presented, the expressive landscape is contoured in a fashion that already is far from that of normal science. The court may not see as helpful, common modes of scientific discourse (carefully qualified conclusions, probabilistic conclusions, acknowledging the possible validity of other points of view, and so forth). The courtroom does not want normal science nor is it particularly useful to the triers of fact. Complaints that LGS1 distorts the discourse of science are therefore misplaced at the outset. If that is the problem, then no scientific testimony would be presented. The question then is whether there is something special about the kinds of departures from normal science caused by LGS1.

The *Daubert* decision requires trial courts to address the questions of reliability and relevance, thus returning to the question of whether the actual scientific methods used were relevant and reliable. So we appear to have returned to our starting point. But this, too, is more problematic than it appears. If this means that the actual techniques used (e.g., chromatography or logistic regression) are in keeping with what is generally considered proper and acceptable, *Daubert* reverts to the Frye standard—that the methods or scientific principles used are “generally accepted” by the scientific community. If on other other hand, it means that the courts should address the more difficult “demarcation problem” of separating science from pseudoscience, judges will find themselves over their heads in the deep waters of controverted epistemology. As we have seen, it is difficult even to discern what methodologic or epistemologic criteria could separate litigation-generated science from other kinds of science.

## Disclosure as a Response to the Question of Bias

Bias of the type Judge Kosinski alleges is a problem that goes beyond the courtroom. Biomedical journals have faced the problem of conflict of interest for a long time. Many journals do not require disclosure of conflicts of interest, but now more than 600 have agreed to follow the manuscript requirements of the International Committee for Medical Journal Editors (2006) on this subject. Authors submitting articles to these journals must disclose all relationships that might involve the appearance of a conflict of interest, and they must disclose study funding. Journal editors will publish this information if they think it might be important for readers. Articles submitted to the journals should be accompanied by signed statements by authors stating that they control the data, analysis, the writing of reports, and submission for publication. Authors must describe any involvement of sponsors in any of these aspects of the study. Editors may include this information if they publish a submitted paper and may also use the information as an input into the decision to accept or reject a submission.

Academic journals address financial conflicts of interest through disclosure, which alerts editors, reviewers, and readers to the potential for bias where conflicts exist. This can lead to greater scrutiny of studies that may be affected by such bias. Disclosure is not a panacea. Disclosure and scrutiny will not necessarily prevent systematic distortions of the scientific literature, even when individual studies are conducted using appropriate methods of data collection and analysis. Sponsors with control over publication can decide which studies to submit, possibly choosing preferentially to submit favorable studies and thus biasing the overall literature on safety or efficacy (Blumenstyk 2003; Dickersin et al. 1992; Easterbrook et al. 1991; Goozner and DelViscio 2004). Still, disclosure does allow readers (or jurors) to engage in greater scrutiny when conflict of interest is present.

## Where Does That Leave Us?

LGS1 is part of the class of research studies involving possible conflicts of interest. The larger set, in which LGS1 is included, also comprises studies of safety and efficacy financed by companies that rely on such studies to obtain licenses to sell their products or that rely on them to promote sales, prelitigation studies financed by parties for product research and development, regulatory science done to influence standards, and scientific work done for criminal litigation, such as fingerprints and handwriting, as well as accident reconstruction. All these can raise questions about possible bias for researchers when they work for any party that has an interest in the outcome of the studies.

This does not mean there are no differences between LGS1 and LGS2 studies. LGS1 can be designed to be more pertinent to a specific case than preexisting science is likely to be. This, however, is not necessarily a defect. The upside of relevance is that studies can be designed to provide data that directly address the legal issues, for example, causation. Indeed, such evidence is often lacking when litigation commences, and case-specific fact-finding can be more efficient precisely because it is more targeted. Other studies of a particular health problem may have been done using populations that differ in some important aspect from the plaintiffs in a toxic tort suit, but litigation-generated studies, of outcome or exposure or both, can be done using a population of which the plaintiff is a member. Because they are more relevant to the case being adjudicated, they can provide better evidence than other studies might. On the other hand, because they are done under the shadow of a conflict of interest, the court may be concerned they are biased. Judge Kosinski’s decision asserted that the resulting biases were always and necessarily of a special nature. It is this contention we have addressed—and questioned—here.

Where does this leave us? We think the courts are rightly advised to be wary of LGS1. However, there seem no strong reasons to treat this conflict of interest differently from other relevant conflicts of interest. Drawing a bright line at the moment litigation begins may be convenient for the court, but it does not serve the interests of justice. And because justice is a primary goal of the legal system, it would seem unwise to construct an unfairly high and one-size-fits-all evidentiary hurdle for science involving conflicts of interest (Jasanoff 2005). In the adversarial and many other settings, such science would involve evidence that is almost certainly relevant. But partial application to only some instances of conflict, for example, the exclusion of evidence about causation in toxic tort cases, potentially creates imbalances. The consequences of evidentiary exclusion differ for plaintiffs and defendants because plaintiffs have the initial burden of presenting evidence. If neither side produces adequate evidence to support its contentions, the plaintiff loses. Excluding defense expert testimony makes defending a case more difficult. Excluding plaintiffs’ expert testimony makes bringing a case impossible.

The focus on excluding postlitigation science affects plaintiffs and defendants unequally because of this burden and because the parties have unequal resources and opportunities to generate prelitigation science. What Henry and Conrad (2008) have stated with respect to regulatory science would seem to hold, as well, for assuring fairness in the trial setting:

In effect, Congress and the courts have determined that in an open, democratic society that is administered by a bureaucracy that is required to act fairly and rationally, it is important that agencies allow interested or affected persons to provide information to them, and fairly consider that information.

The courts have one method of dealing with conflict of interest not available to scientific journals and their readers: cross-examination. With their own experts as consultants, attorneys have become adept at deconstructing the research and arguments of opposing experts. They also can point out to the jury when research presented by an expert has been funded by and controlled by a party to the litigation. Some may consider allowing the jury to hear such evidence an abdication of judicial responsibilities under *Daubert* (Anderson et al. 2001). However, if a study appears to be relevant and there is no specific evidence that it was improperly designed or executed, suspicions generated by conflicts of interest are within the scope of jury competence. Juries may sometimes find it difficult to understand complex science, but most understand conflicts of interest and can judge the science presented to them with that in mind.

The problems with litigation-generated science are not special. On the contrary, they are very general and apply to much or most science that is relevant and reliable in the courtroom setting.
